# Effects of Vestibular Rehabilitation on Fatigue and Activities of Daily Living in People with Parkinson's Disease: A Pilot Randomized Controlled Trial Study

**DOI:** 10.1155/2020/8624986

**Published:** 2020-09-10

**Authors:** Amirabas Abasi, Parvin Raji, Joseph H. Friedman, Mohammad-Reza Hadian, Reza Hoseinabadi, Somaye Abbasi, Ahmadreza Baghestani

**Affiliations:** ^1^Department of Occupational Therapy, School of Rehabilitation, Tehran University of Medical Sciences, Tehran, Iran; ^2^Department of Neurology, Warren Alpert School of Medicine at Brown University, Providence, RI, USA; ^3^School of Rehabilitation, Brain and Spinal Injury Research Center (BASIR), Institute of Neuroscience, Tehran University of Medical Sciences (TUMS), International Campus TUMS, Tehran, Iran; ^4^Department of Audiology, School of Rehabilitation, Tehran University of Medical Sciences, Tehran, Iran; ^5^Department of Biostatistics, School of Allied Medical Sciences, Shahid Beheshti University of Medical Sciences, Tehran, Iran

## Abstract

One of the most disabling nonmotor symptoms in persons with Parkinson's disease is fatigue, which can decrease the quality of life by restricting the function and activities of daily living (ADL). Nonetheless, sufficient evidence for treating fatigue, including drug or nondrug treatment, is not available. In this study, we evaluated the probable effects of vestibular rehabilitation on fatigue and ADL in patients with Parkinson's disease. *Methods*. This was a single-blind clinical trial study in which patients with Parkinson's disease voluntarily participated based on the inclusion and exclusion criteria. The patients were randomly assigned to the case and control groups. The case group received 24 sessions of vestibular rehabilitation protocol, and conventional rehabilitation was performed in the control group (i.e., 3 sessions each week, each lasted about 60 minutes). Both groups were also given fatigue management advice. Fatigue was measured by the Parkinson Fatigue Scale (PFS) and the Modified Fatigue Impact Scale (MFIS). ADL was measured by the Functional Independence Measure (FIM). All changes were measured from the baseline at the completion of the intervention. *Results*. Both fatigue (*P* ≤ 0.001) and ADL (*P* ≤ 0.001) improved significantly more in the vestibular intervention group than in the control one. *Conclusion*. Vestibular rehabilitation may improve fatigue and ADL and therefore can be used as an effective intervention for patients with Parkinson's disease, which was also found to be well tolerated.

## 1. Introduction

Fatigue is one of the most common nonmotor symptoms of Parkinson's disease (PD) [1, 2]. It causes disability and plays a significant role in reducing the quality of life of PD patients [3].

There are various types of fatigue. Krupp and Pollina noted that fatigue can be due to neurological, somatic, or mental dysfunction and is perceived as an excessive sense of exhaustion, frustration, or lack of energy by the patient [4]. Fatigue is a sense of exhaustion that is not the result of excessive exercise, medication side effects, or other medical or psychiatric diseases. It differs from usual, nonpathological fatigue, in which it exceeds previously experienced fatigue by a great deal and feels different from the patients. It is a chronic state which is not initiated by extra effort or exercise [5]. Nonetheless, there are insufficient data on the treatment of fatigue in PD. In a systematic review of fatigue in patients with Parkinson's disease, Siciliano et al. reported that fatigue may decrease working time, social participation, sports participation, and personal activities [6, 7]. Therefore, fatigue is considered as an important complaint that requires attention and better management for patients with Parkinson's disease.

Central sensory-motor integration (i.e., somatosensory, visual, and vestibular systems) is important for efficient postural control and might be affected by the sense of fatigue in patients with Parkinson's disease [8, 9]. Somatosensory, visual, and vestibular systems as the main components of central sensory-motor integration had been positively triggered by vestibular rehabilitation. Vestibular rehabilitation is a training program for patients with postural control disorders which are thought to improve postural stability and performance through compensatory and adaptation mechanisms, by the repetition of tasks [10].

Reviewing the literature, we saw no study to investigate the effect of vestibular rehabilitation exercises on fatigue in PD; however, Hebert et al. provided evidence about the effects of vestibular rehabilitation on multiple sclerosis-related fatigue and upright postural control [11]. Besides, Tramontano et al. reported that vestibular rehabilitation had positive effects on balance, fatigue, and ADL in highly disabled multiple sclerosis people [12]. Also, this type of training had improved dizziness and balance in patients with Parkinson's disease [13]. An improvement in the activities of daily life, gait velocity, and balance and a reduction in the risk of falls were reported by Rossi-Izquierdo. Consequently, we hypothesized that vestibular rehabilitation exercises could improve both fatigue and the ability to participate in activities of daily life in PD.

## 2. Methods

This was an interventional pilot study using a single-blind clinical design. The eligible subjects of this study were a convenience sample of patients who had been referred to Rasoul-e-Akram and Imam Khomeini hospitals. Based on the pilot results and the inclusion and exclusion criteria, 24 patients were randomly assigned to two equal groups: twelve patients to the control group and twelve patients to the case group. All measures were performed in the first session and immediately after the last session by a senior occupational therapist (M. Sc. degree) who was blinded to the intervention; all investigations were performed in the medicine phase of “on” (i.e., when medicine had its maximum effect [14]). The study was carried out between March 2018 and December 2019.

### 2.1. Standard Protocol Approvals

Eligible subjects were enrolled after signing an informed consent form and the study was approved by the Iranian Registry Center of the Clinical Trials (IRCT201709123551N6) and the Ethical Committee of Tehran University of Medical Sciences (IR.TUMS.FNM.REC.1396.3210).

### 2.2. Sample Size and Randomization

A pilot, open-label trial enlisted 5 subjects; calculations by considering *α* = 0.05 and *β* = 0.20 and mean changes of the Parkinson Fatigue Scale showed that 12 patients were required in each group. Twenty-four patients were randomly allocated to one of the two groups, and randomization was conducted based on encoded envelopes; each code was assigned to a patient who agreed to participate in the study [15].

### 2.3. Participants

The inclusion criteria were as follows: Hoehn and Yahr (H&Y) stage 1–4. The stages are defined as follows: stage 1: the patients had unilateral involvement only, usually with minimal or no functional disability; stage 2: bilateral or midline involvement, without impairment of balance; stage 3: bilateral disease: mild to moderate disability with impaired postural reflexes and physically independent; and stage 4: severely disabling disease, still able to walk or stand unassisted [16]. Also, to assure vestibular dysfunction in participants, videonystagmography (VNG) tests were performed by a senior audiologist; therefore, patients must avoid caffeine (coffee, tea, and cola) after midnight before testing. Moreover, they must discontinue all medications 48 hours before the test except “maintenance” medication for their heart, blood pressure, diabetes, or seizures and any medications deemed by their physician to be necessary [17]. Having a score ≥45 for the Modified Fatigue Impact Scale (MFIS) [18, 19] and a score >23 on the Mini-Mental State Examination (MMSE) [20].

**Figure 1:**
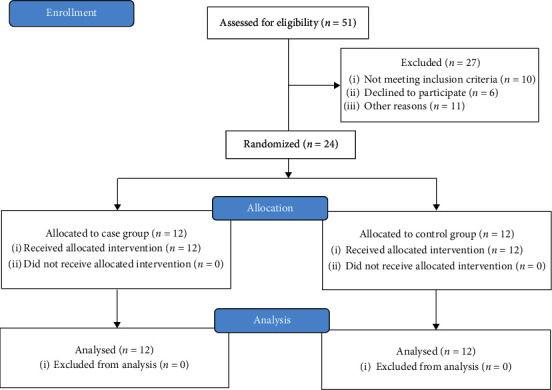
Study design schematic.

Patients with severe daytime sleepiness, depression, and apathy symptoms were excluded based on their historical medication [7]. Additionally, patients with diabetes, significant osteoarthritis, and osteoporosis, particularly in the lower limbs, or other neurological diseases were not participated[10]. Patients with lack of cooperation during the assessments and exercise protocols, patients with lack of interest in participating in the second stage of the assessments after the intervention, and patients who had any change in their medication during the intervention were excluded from the study (Figure 1 for details).

### 2.4. Assessment Tools

The Parkinson Fatigue Scale (PFS) contains 16 questions: 7 questions related to tiredness and the effect of physical fatigue and 9 questions that evaluate the effect of fatigue on performance and daily activities. As mentioned, the questionnaire was completed by the subjects, and the score for each question ranged from 1 (definitely disagree) to 5 (definitely agree) [21, 22].

As the Parkinson Fatigue Scale (PFS) Persian format has no related classification cutoff score, fatigue was measured by the Modified Fatigue Impact Scale (MFIS) as well. The MFIS is a valid measure with 21 self-report items and three subscales: physical (9 items), cognitive (10 items), and psychosocial (2 items); each item was scored from 0 (never) to 4 (almost always), and a total score is 84 [19, 23].

The Functional Independent Measure (FIM) questionnaire is used to evaluate the daily activities. This questionnaire contains 18 items (13 items related to motor skills and 5 items related to the cognitive one). Each item has 7 ratings in which 7 means complete independence in activities and 1 means complete dependence, requiring maximum help in performing daily activities [24, 25].

### 2.5. Interventions

Vestibular rehabilitation exercises were performed for about 60 minutes at each of the 24 sessions, three days a week in the case group. The vestibular rehabilitation exercises included the following exercises: exercises on a trampoline; exercises on a firm surface, foam, and a balance board with open and closed eyes; head movements to the sides, upward, and downward; throwing and catching a ball in which alterations were made in the center of gravity; walking exercises and simultaneous movement of the head; and moving a ball in hands from side to side. Oculomotor exercises took about 10 minutes of the treatment session [26–28] (Appendix 1).

The control group was treated as follows: five minutes of warm-up exercises (including slow walking), stretching exercises (scapular muscles, pelvic flexors, hamstrings, and gastrocnemius) for 15 minutes [29], body rotation (body rotation pattern) for 15 minutes as well as suggestions for fatigue management based on best practice guidelines [30]. The patients were given time to rest during the training whenever they felt tired.

### 2.6. Statistical Analysis

All statistical analyses were performed by a senior biostatistician. Independent *t*-test and chi-square tests were used to determine the significance level of data (*P* < 0.05 was considered statistically significant) about the inclusion criteria between the two groups (age, gender, disease duration, H&Y, PFS, MFIS, and FIM). The paired *t*-test was used (due to normality of data distribution) for comparing the scores before and after the intervention in both case and control groups.

## 3. Results

The complete information about the demography and inclusion criteria is listed in Table 1. As it could be seen, twelve patients were assigned to the case group (mean age 63.16, with 4 subjects in H&Y stage III and 8 in stage II) and twelve patients in the control group (mean age 63.08, with 5 subjects in stage III and 7 in stage II). There was no significant difference between the two groups in terms of inclusion criteria. There was no significant difference between the case and control groups (*P* ≤ 0.634) in terms of fatigue, the total score of FIM (*P* ≤ 0.402), and the MFIS at baseline (*P*=0.209).

The case group showed a significant difference in PFS (*P* < 0.001); the control group did not change significantly (*P* = 0.083). Total score of FIM, score of motor, and cognitive outcomes of FIM in the case group were respectively *P* < 0.001, *P* < 0.001, and *P* = 0.076. Also, FIM scores in the control group were *P* = 0.056, *P* = 0.089, and *P* = 0.339, respectively (Table 2).

The significant difference was found in PFS for the case group (*P* < 0.001) compared with the control group. No significant difference was found in FIM cognitive outcome (*P*=0.106), but the motor outcome score and the total FIM differences were significant (*P* < 0.001 and *P* < 0.001) between the case group and the control group (Table 3).

## 4. Discussion

This single-blind pilot study demonstrated significantly greater improvement in both fatigue and ADL in the case than in the control group.

The primary outcome variable in this study was the change of fatigue which, in the case group, was improved by 12.58 in PFS compared to 1.58 in the control group (*P* < 0.001). To the best of our knowledge, no study has been published on the possible effects of vestibular rehabilitation on fatigue in patients with Parkinson's disease. Therefore, we cannot compare our results with other studies; however, Hebert et al. investigated the effects of vestibular rehabilitation on fatigue in multiple sclerosis patients. They found that vestibular rehabilitation produced significant improvement in fatigue MFIS (*P* < 0.001) [11].

Addressing the underlying mechanism of fatigue improvement was not the purpose of our study, but our findings may provide a theoretical insight into the probable role of neuroplasticity as an explanation for the beneficial effects [31]. Previous studies suggested that there was an uncertain relationship between fatigue and stage of disease and duration as well as motor symptoms in PD [7, 32]. Therefore, neurophysiological explanations for improvement in fatigue after vestibular therapy have not been considered [5]. Our study suggests there may be such a connection.

Vestibular rehabilitation had significant benefits for the activities of daily living (FIM). Our findings agreed with Rossi-Izquierdo et al. who reported that vestibular exercise in patients with Parkinson's disease improved ADL, with faster walking, improved balance, and reduced the risk of falls [10]. Balance exercises in the vestibular rehabilitation protocol played a major role in providing appropriate postural control during standing and walking [11]. Also in a preliminary study, Bonnì et al. had provided evidence about the neurophysiological effects of blindfolded balance training in patients with Parkinson's disease, which is a very similar training based on the stimulation of vestibulospinal reflex; their findings supported that probably when the supplementary motor area (SMA) is stimulated by vestibular training in dynamic conditions without visual afferents, it may produce a sensory-motor gain and contribute to improving the anticipatory postural adjustment (APA), leading to an efficient and quick gait rehabilitation [33]. Possibly a lower amount of energy is required for maintaining balance after vestibular therapy, which might affect the improvement of the total and the motor outcome of FIM.

As mentioned earlier in the Method section, the vestibular rehabilitation comprises oculomotor exercises which took about 10 minutes of each treatment session. Oculomotor exercises may play an important role in neuromuscular reorganization [34]. As oculomotor pathways are thought to be impaired in PD [35], which is reflected in worsened postural control [36], so these exercises might improve the coordination of the head, torso, and pelvic girdle movement during walking and help to maintain the postural stability [37, 38].

## 5. Conclusions

The results of this study showed the benefits of vestibular rehabilitation for fatigue and activities of daily living in patients with Parkinson's disease. Why vestibular rehabilitation helped more than standard therapy is unclear but may involve novel neuromuscular reorganization mechanisms. Also, this type of intervention is low cost, safe, and practical in clinics and homes with minimal facilities and therefore can be recommended for both resource-poor and resource-rich societies.

## 6. Study Limitation

A small number of patients did not provide the possibility of generalization. Furthermore, for the future study, a follow-up phase may provide a reasonable outcome of the maintenance of the effects of vestibular rehabilitation exercises in patients with Parkinson's disease.

## Figures and Tables

**Table 1:** Demographic data, mean score, and standard deviation of the patients (*n* = 24).

Variable	Case group (*n* = 12)	Control group (*n* = 12)	Significant level
Age (years)	63.16 (8.05)	63.08 (9.49)	0.982
Male/female	4/8	6/6	0.408^α^
Time since diagnosis (years)	3.33 (1.55)	3.75 (1.60)	0.525
H&Y grade II/III	4/8	5/7	0.178^α^
BMI	25.30 (3.18)	25.07 (4.19)	0.884
MMSE (score)	24.81 (0.98)	25.23 (1.01)	0.549
PFS	55.66 (7.22)	54.16 (7.98)	0.634
FIM total score	113.33 (6.82)	110.83 (7.48)	0.402
MFIS total score	48.41 (3.44)	46.75 (2.37)	0.209

Mean scales and standard deviation. Independent *t*-test and chi-square test (*α*) were used to evaluate the level of significance. H&Y: Hoehn and Yahr scale. BMI: body mass index. MMSE: Mini-Mental State Examination.

**Table 2:** Fatigue and functional independence in daily activities before and after the interventions (at the end of 24^th^ session).

Variable	Case group (*n* = 12)	Control group (*n* = 12)	Effect size (*r*)	Effect size (*d*)
*MFIS*				
Before intervention	48.41 (3.44)	46.75 (2.37)	0.85	3.25
After intervention	32.33 (7.60)	46.16 (2.69)		
Changes	16.08 (6.65)	0.58 (1.08)		
95% CI	11.85 to 20.31	−0.10 to 1.27		
*P*	<0.001	0.089		
*PFS*				
Before intervention	55.66 (7.22)	54.16 (7.98)	0.79	2.63
After intervention	43.08 (7.84)	52.58 (6.47)		
Changes	12.58 (5.16)	1.58 (2.87)		
95% CI	9.30 to 15.86	−0.24 to 3.40		
*P*	<0.001	0.083		
*FIM cognitive outcome*				
Before intervention	32.58 (2.42)	31.16 (2.97)	0.33	1.62
After intervention	33.50 (2.27)	31.25 (2.95)		
Changes	0.91 (1.62)	0.08 (0.28)		
95% CI	−1.94 to 0.11	−0.26 to 0.10		
*P*	0.076	0.339		
*FIM motor outcome*				
Before intervention	81.50 (5.71)	79.75 (6.09)	0.77	2.42
After intervention	88.91 (2.96)	31.25 (2.95)		
Changes	7.41 (3.84)	0.58 (1.08)		
95% CI	−9.86 to −4.97	−1.27 to 0.10		
*P*	<0.001	0.089		
*FIM total score*				
Before intervention	113.33 (6.82)	110.83 (7.48)	0.81	2.84
After intervention	122.41 (4.79)	111.58 (8.16)		
Changes	9.08 (3.96)	0.75 (1.21)		
95% CI	−11.60 to −6.56	−1.52 to 0.02		
*P*	<0.001	0.056		

Mean and standard deviation scales before and after the intervention; the mean and standard deviation of changes; 95% CI: 95% confidence interval. *P*: level of significance before and after the intervention for both groups. Effect size (*r*): numeric difference in change of outcome measure among groups. Effect size index (*d*): Cohen *d* standard effect size index.

**Table 3:** Comparison between the case and control groups in PFS and FIM tests.

Variable	Comparison between intervention and control group
*MFIS*	
*T*	7.965
*P*	<0.001
Effect size (*r*)	0.92
Effect size (*d*)	4.80
*PFS*	
*T*	6.451
*P*	<0.001
Effect size (*r*)	0.88
Effect size (*d*)	3.89
*FIM cognitive outcome*	
*T*	−1.753
*P*	0.106
Effect size (*r*)	0.46
Effect size (*d*)	−1.05
*FIM motor outcome*	
*T*	−5.921
*P*	<0.001
Effect size (*r*)	0.87
Effect size (*d*)	−3.57
*FIM total score*	
*T*	−6.860
*P*	<0.001
Effect size (*r*)	0.90
Effect size (*d*)	−4.13

*T* is a statistic of independence *t*-test and *P* value (*P*) shows the level of significance before and after intervention for both groups. Effect size (*r*): numeric difference in change of outcome measure among groups. Effect size index (*d*): Cohen *d* standard effect size index.

## Data Availability

All data used to support the results of this study are included in the article.

## Appendix

### Vestibular Rehabilitation Protocol

Static Position: Standing and Half Kneeling
  - Firm surface
  - Foam surface
  - Trampoline
  - Tilt board

Each item was performed with open and closed eyes and head rotations to each side as well as throwing and catching a ball.

Dynamic Position: Walking
  - Tandem gait forward and backward
  - Walking with a ball in hand and turning side to side as well as tracking the ball
  - Stop and start walking, rotating 180 degrees in the direction as well as standing on one leg while it was ordered
Oculomotor Training
  - Saccade: rapid eye movement between 2 objects in 4 directions (horizontal, vertical, and 2 diagonal directions)
  - Smooth pursuit: tracking an object in 4 directions, while the head is stable
  - Vestibuloocular movements: rotating the head side to side, up, and down, while gazing at a subject
